# Fe_5_Cu_5_V_30_Ti_30_Nb_30_ High-Entropy Alloy Films as Cr- and Al-Free Sensing Layers for Thin-Film Strain Gauges in High-Pressure Hydrogen

**DOI:** 10.3390/ma19153292

**Published:** 2026-08-03

**Authors:** Wanliang Zhang, Kaiyu Zhang, Chengshuang Zhou, Lin Zhang

**Affiliations:** Institute of Material Forming and Control Engineering, Zhejiang University of Technology, Hangzhou 310014, China; zhwl0525@gmail.com (W.Z.); 2111825057@zjut.edu.cn (K.Z.); zhoucs@zjut.edu.cn (C.Z.)

**Keywords:** high-entropy alloy film, thin-film strain gauge, high-pressure hydrogen, zero drift, BCC structure

## Abstract

A Fe_5_Cu_5_V_30_Ti_30_Nb_30_ high-entropy alloy film was designed as a Cr- and Al-free metallic sensing layer for thin-film strain gauges in high-pressure hydrogen environments. CALPHAD calculations predicted a BCC/B2-type phase field, while XRD, EBSD and GIXRD results supported a BCC-type structure without direct confirmation of long-range B2 ordering. Fe_5_Cu_5_V_30_Ti_30_Nb_30_ films deposited on Si reference substrates at 150 and 300 W retained broad BCC-type diffraction features. The 300 W film showed a more continuous cross-sectional morphology, good metallic conductivity and a comparable nanomechanical response with slightly higher hardness. Device-level tests were then performed using Cr/AlN/Fe_5_Cu_5_V_30_Ti_30_Nb_30_ TFSGs on 316L stainless-steel substrates. The devices exhibited average absolute apparent zero shifts of 16.08 με in 12 MPa N_2_ and 17.79 με in 12 MPa H_2_, with an additional H_2_-associated apparent response of only 1.71 με. Static tensile tests in 12 MPa H_2_ confirmed a linear strain response with a gauge factor of 1.72 ± 0.01.

## 1. Introduction

Hydrogen has become an important energy carrier for the storage and transport of clean energy [1,2,3,4,5,6]. In high-pressure hydrogen systems, accurate stress and strain monitoring is essential for evaluating the structural safety of pressure vessels, valves and pipelines [7,8,9,10]. Resistance strain gauges are widely used for this purpose. However, their electrical output can be disturbed by hydrogen ingress. Hydrogen atoms may enter the metallic sensing layer and change its resistance. This can lead to zero drift, creep and signal instability under high-pressure hydrogen environments [11,12,13].

Thin-film strain gauges (TFSGs) provide a promising solution to this problem. They can be directly fabricated on metallic substrates by physical vapor deposition. This avoids the use of organic adhesives and improves the interfacial stability between the sensor and substrate. In previous work [14], Cr/AlN/FeCrAl TFSGs were developed for high-pressure hydrogen environments. The FeCrAl sensing layer showed low zero drift and good resistance stability in 12 MPa H_2_. Nevertheless, FeCrAl contains Cr and Al, which are prone to forming passive oxide films [15]. These oxides can reduce surface wettability and make electrode welding more difficult during device fabrication [16,17,18]. Therefore, it is necessary to develop a new metallic sensing layer that maintains hydrogen resistance while reducing the processing limitations caused by Cr- and Al-containing passive films.

High-entropy alloys (HEAs) offer a useful strategy for designing stable metallic sensing layers [19,20]. It should be noted that HEAs can be classified using either composition-based or entropy-based criteria. In the present work, the term HEA is used according to the classical composition-based definition, namely alloys containing at least five principal metallic elements, with each element having an atomic fraction between 5 and 35 at.% [21]. This definition has been widely used in the HEA field, although some non-equiatomic alloys satisfying the composition-based criterion may have ideal configurational entropy values near the medium-entropy/high-entropy boundary. Compared with conventional binary or ternary alloys, multicomponent HEAs also provide a broader compositional design space. Their phase formation can be preliminarily screened using empirical parameters, such as valence electron concentration (VEC), atomic size mismatch (δ), mixing enthalpy (ΔH_mix_), mixing entropy (ΔS_mix_), electronegativity difference (Δχ) and the Ω parameter [22,23,24,25]. CALPHAD calculations can further predict phase stability and guide alloy composition design before experimental fabrication [26,27].

Hydrogen interactions with high-entropy or multi-principal-element alloys are complex and strongly dependent on alloy composition and microstructure. Refractory HEAs have also been studied in the context of hydrogen embrittlement, where hydrogen effects were found to depend strongly on phase constitution, local chemical environment and deformation behavior [28,29,30]. Previous studies have shown that hydrogen dissolution, diffusion and trapping in HEAs can be affected by local chemical complexity, lattice distortion and the distribution of interstitial environments [31,32]. However, these effects should not be assumed to be universally beneficial for hydrogen resistance. In particular, some BCC Ti-, V- and Nb-containing refractory HEAs have also been investigated as hydrogen-sorption or hydride-forming materials [33,34,35]. Therefore, in the present work, the Fe_5_Cu_5_V_30_Ti_30_Nb_30_ film is evaluated mainly from the viewpoint of device-level electrical stability in high-pressure gas environments, rather than from direct hydrogen-transport measurements.

Based on these considerations, a compositionally defined non-equiatomic Fe_5_Cu_5_V_30_Ti_30_Nb_30_ HEA was designed in this work as a new sensing-layer material for high-pressure hydrogen TFSGs. The alloy composition was selected using the classical composition-based HEA criterion, empirical parameters for BCC-type solid-solution formation, and CALPHAD phase-stability prediction. A bulk HEA target was prepared by vacuum arc melting and homogenization. HEA films were then deposited by magnetron sputtering at different powers. Their phase structure, morphology, composition, electrical resistivity and nanomechanical response were systematically investigated. Finally, the optimized HEA film was used as the sensing layer to fabricate HEAF TFSGs, and its zero-drift behavior was evaluated in a 12 MPa H_2_ environment. This study aims to clarify the relationship among alloy design, sputtering power, film structure and hydrogen-environment electrical stability. Fe_5_Cu_5_V_30_Ti_30_Nb_30_ HEA films were designed as Cr- and Al-free metallic sensing layers to replace the FeCrAl sensing layer in the Cr/AlN/metallic-sensing-layer TFSG architecture.

## 2. Materials and Methods

### 2.1. CALPHAD Calculation and Empirical Parameter Evaluation

The nominal composition of the designed high-entropy alloy was Fe_5_Cu_5_V_30_Ti_30_Nb_30_. The empirical phase-formation parameters, including VEC, ΔH_mix_, ΔS_mix_, δ, Ω, Δχ and T_m_, were calculated according to commonly used high-entropy alloy design equations. These parameters were used to preliminarily evaluate the tendency for BCC solid-solution formation. Thermodynamic equilibrium calculations were performed using Thermo-Calc 2023B with the THEA6 high-entropy alloy database. The equilibrium phase fractions were calculated as a function of temperature to predict the phase stability of the designed alloy.

### 2.2. Preparation of the Fe_5_Cu_5_V_30_Ti_30_Nb_30_ Bulk HEA Target

The Fe_5_Cu_5_V_30_Ti_30_Nb_30_ bulk alloy and sputtering target were prepared by vacuum arc melting. High-purity elemental metals with purities higher than 99.9 wt.% were used as raw materials (Beijing Zhongke Yannuo New Materials Technology Co., Ltd., Beijing, China). The elements were accurately weighed according to the nominal composition and manually mixed in an argon-filled glove box. The mixed metals were then placed in a water-cooled copper crucible.

Arc melting was carried out in a high-vacuum non-consumable tungsten-electrode arc furnace (DHL-400, Zhengzhou Chengyue Scientific Instrument Co., Ltd., Zhengzhou, China). Before melting, the chamber was evacuated to a base pressure lower than 5.0 × 10^−3^ Pa and then backfilled with high-purity argon. The alloy ingot was flipped and remelted at least six times to improve chemical homogeneity. After melting, the ingot was cooled to room temperature in the furnace and then machined into a sputtering target.

The as-cast alloy was homogenized using a programmed heat-treatment process. The sample was first heated to 600 °C at 10 °C min^−1^ and held for 20 min. It was then heated to 1200 °C and held for 6 h to promote elemental diffusion and chemical homogenization. After homogenization, high-purity inert gas was introduced into the furnace (VHB-335H, Shenyang Jiayu Vacuum Technology Co., Ltd., Shenyang, China), and forced convection cooling was applied to cool the sample to room temperature. The homogenized alloy was used for subsequent structural characterization and thin-film deposition.

### 2.3. Deposition of HEA Films and Fabrication of HEAF TFSGs

Fe_5_Cu_5_V_30_Ti_30_Nb_30_ high-entropy alloy films were deposited on single-side polished Si wafers using a JGP-450 vacuum magnetron sputtering ion-plating system (Shenyang Sky Technology Development Co., Ltd., Shenyang, China). The films were prepared at two sputtering powers, 150 and 300 W, and are denoted as HEAF-150W and HEAF-300W, respectively. These films were used for structural, compositional, electrical and nanomechanical characterization. The Si wafers were used as flat reference substrates for preliminary thin-film characterization and for comparing the effect of sputtering power under controlled deposition conditions. Similar use of Si-based reference substrates has been reported in thin-film strain gauge material studies, for example, in the characterization of CrN and TiAlN_x_O_γ_ sensing films [36,37]. However, the growth behavior of Fe_5_Cu_5_V_30_Ti_30_Nb_30_ films on Si may not be identical to that on the 316L/Cr/AlN multilayer structure because substrate roughness, surface chemistry, interfacial energy and residual stress can influence film morphology and texture. Therefore, the Si-substrate film data are used in this work only for comparative screening of deposition conditions, while the final sensing performance is evaluated using actual Cr/AlN/Fe_5_Cu_5_V_30_Ti_30_Nb_30_ TFSGs fabricated on 316L stainless-steel substrates. Before deposition, the chamber was evacuated to 3.0 × 10^−3^ Pa. The working pressure was maintained at 0.8 Pa with an Ar flow rate of 25 sccm. The substrate temperature was kept at room temperature, approximately 23 °C. A substrate bias voltage of 90 V was applied. The deposition time was 40 min. Before film deposition, the HEA target was pre-sputtered for 10 min to remove surface oxides and adsorbed contaminants.

The HEAF thin-film strain gauges were fabricated with a Cr/AlN/Fe_5_Cu_5_V_30_Ti_30_Nb_30_ multilayer structure on 316L stainless-steel substrates. The Cr layer served as a transition/buffer layer, the AlN layer served as an electrical insulating layer, and the Fe_5_Cu_5_V_30_Ti_30_Nb_30_ HEA film was used as the metallic sensing layer. In this work, the term “Cr- and Al-free” refers specifically to the Fe_5_Cu_5_V_30_Ti_30_Nb_30_ metallic sensing layer rather than to the entire TFSG device. The complete device still contains the Cr transition layer and the AlN insulating layer. These two auxiliary layers were not used as the resistive sensing element and were not intentionally connected to the external measurement circuit. The resistance signal was collected from the patterned Fe_5_Cu_5_V_30_Ti_30_Nb_30_ HEA sensing grid.

It should be noted that, although the Cr and AlN layers do not directly serve as the electrical sensing element, they may affect strain transfer from the 316L substrate to the sensing layer and the interfacial stress state. Therefore, the Cr and AlN layers were prepared using identical deposition parameters for all TFSGs. The device structure, mask-patterning process and lead connection method were consistent with those used for the previously reported FeCrAl TFSGs [14]. In the HEAF TFSGs, only the metallic sensing layer was replaced by the Fe_5_Cu_5_V_30_Ti_30_Nb_30_ HEA film. Based on the film characterization results, the 300 W HEA film deposited at room temperature was selected as the sensing layer for zero-shift and strain response evaluation in high-pressure hydrogen.

### 2.4. Characterization, Zero-Drift Test, and Strain Response Test of HEAF TFSGs

The phase structure of the bulk HEA target was characterized by X-ray diffraction. The phase structures of HEAF-150W and HEAF-300W films were analyzed by grazing-incidence X-ray diffraction (GIXRD, SmartLab 9 kW, Rigaku Corporation, Akishima, Tokyo, Japan). The grazing incidence angle was fixed at 1° to reduce the influence of the Si substrate.

The surface and cross-sectional morphologies of the films were observed using field-emission scanning electron microscopy (FESEM, ZEISS ΣIGMA, ZEISS, Oberkochen, Germany). The elemental compositions of the films were measured by energy-dispersive X-ray spectroscopy (EDS) on the cross-sections. The phase distribution and crystallographic orientation of the homogenized Fe_5_Cu_5_V_30_Ti_30_Nb_30_ bulk alloy were examined by electron backscatter diffraction (EBSD). The EBSD specimen was vibratory polished with a 0.02 μm colloidal silica suspension for 4 h. EBSD data were collected using an FEI NOVA 450 scanning electron microscope (FEI Company, Hillsboro, OR, USA) equipped with an Oxford Instruments Symmetry detector (Oxford Instruments NanoAnalysis, High Wycombe, UK) at an accelerating voltage of 20 kV and a step size of 0.5 μm. The data were processed using AZtecCrystal software (version 2.1, Oxford Instruments NanoAnalysis, High Wycombe, UK).

The electrical resistivity of the films was measured at room temperature using a four-probe method with an RTS-8 four-probe tester. Nanoindentation tests were performed using an Agilent G200 nanoindenter (Agilent Technologies, Santa Clara, CA, USA), equipped with a Berkovich diamond tip to evaluate the nanomechanical response of the films. To reduce the substrate effect, the maximum indentation depth was fixed at 200 nm, which was less than one tenth of the film thickness.

The zero-drift performance of the HEAF TFSGs in high-pressure hydrogen was evaluated using the same testing system reported for the FeCrAl TFSGs [14]. The system consisted mainly of a universal tensile testing machine equipped with a high-pressure hydrogen chamber, a static strain indicator and a computer-based data acquisition system. The HEAF TFSGs were exposed to a 12 MPa H_2_ environment, and the indicated strain was recorded during the constant-pressure stage. In this work, only the zero-drift data from the pressure stabilization point to the pressure release point are presented and discussed.

To separate the pressure-induced apparent response from the H_2_-associated electrical response, additional control tests were conducted in 12 MPa N_2_. Three independently fabricated HEAF TFSGs were tested in 12 MPa N_2_ and 12 MPa H_2_, respectively. The pressurization rate, maximum pressure, pressure-holding time, bridge configuration, data-acquisition parameters, and temperature conditions were kept identical for the two gas environments. The complete pressure cycle, including pressurization, pressure holding, depressurization, and recovery, was recorded.

To evaluate the basic strain-sensing performance of the HEAF TFSGs, static tensile tests were further carried out in 12 MPa H_2_ at 23.0 ± 0.3 °C. The tensile specimen was loaded at a constant displacement rate of 0.05 mm min^−1^, and the output signal of the HEAF TFSGs was continuously acquired. The gauge factor was calculated according to:

(1) $$GF=\frac{\Delta R}{\varepsilon R}=(1+2\mu )+\frac{d\rho /\rho }{\varepsilon }$$

where R, ΔR, ε, μ, and ρ are the initial resistance, resistance change, applied strain, Poisson’s ratio, and resistivity of the sensing film, respectively.

## 3. Results and Discussion

### 3.1. Alloy Design and CALPHAD Prediction

The non-equiatomic Fe_5_Cu_5_V_30_Ti_30_Nb_30_ alloy was designed according to the classical composition-based HEA definition and further screened using commonly used empirical parameters for BCC-type solid-solution formation. The designed alloy contains five principal elements, Fe, Cu, V, Ti and Nb, with atomic fractions of 5, 5, 30, 30 and 30 at.%, respectively. Therefore, it satisfies the composition-based HEA criterion requiring at least five principal metallic elements and 5–35 at.% for each element. As shown in Table 1, the calculated ΔS_mix_ is 11.5 J mol^−1^ K^−1^, corresponding to approximately 1.38R. This value is close to the medium-entropy/high-entropy boundary when a strict entropy-threshold criterion is adopted; therefore, the classification of Fe_5_Cu_5_V_30_Ti_30_Nb_30_ as an HEA in this work is based on the classical composition criterion rather than solely on ΔS_mix_.

The empirical parameters in Table 1 support a tendency toward BCC-type solid-solution formation. The calculated VEC is 5.15, which is below the commonly used BCC-favoring threshold of 6.87. The mixing enthalpy, ΔH_mix_ = −2.69 kJ mol^−1^, falls within the typical solid-solution formation range of −15 to 5 kJ mol^−1^. The atomic size mismatch, δ = 5.29%, is below the commonly used upper limit of approximately 6.5–6.6%, while Ω = 9.46 is much higher than the empirical criterion of Ω ≥ 1.1. In addition, the small electronegativity difference, Δχ = 0.086, suggests a limited chemical mismatch among the constituent elements. These empirical criteria are used only for preliminary alloy screening, and the phase constitution was further evaluated using CALPHAD calculations and experimental characterization.

**Figure 1:**
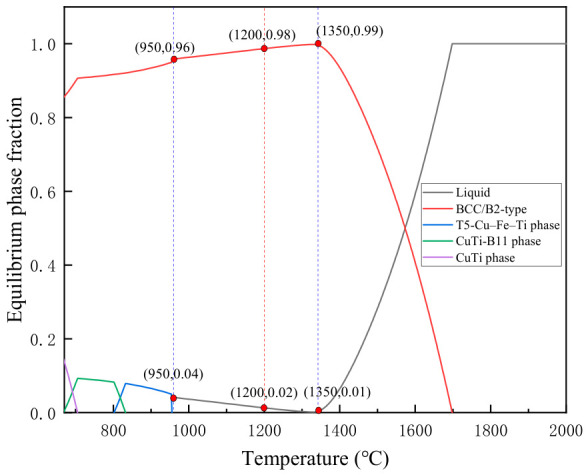
The alloy is predicted to be dominated by a BCC/B2-type phase field near the 1200 °C homogenization temperature.

To further evaluate the phase stability of the designed alloy, equilibrium phase-fraction calculations were performed using the CALPHAD method with Thermo-Calc 2023B and the THEA6 high-entropy alloy database. The calculated phase evolution is shown in Figure 1. The CALPHAD calculation predicts that the phase field labelled as BCC_B2 in the database is dominant over a broad temperature range, with calculated phase fractions of approximately 0.96 at 950 °C, 0.98 at 1200 °C and 0.99 at 1350 °C. In this work, “BCC_B2” is used only as the CALPHAD database phase label. This result suggests that the designed Fe–Cu–V–Ti–Nb alloy has a strong thermodynamic tendency to maintain a BCC-based matrix at elevated temperatures. It should be emphasized that this prediction does not by itself confirm long-range B2 ordering; therefore, the experimentally observed phase constitution is conservatively described as BCC-type in the following sections.

The selected homogenization treatment temperature of 1200 °C lies within the CALPHAD-predicted BCC_B2-labelled stability region. At this temperature, the calculated phase fraction of this BCC-based phase approaches 0.98, while only a small fraction of secondary phase is predicted under equilibrium conditions. Therefore, the 1200 °C homogenization treatment is expected to promote chemical homogenization after vacuum arc melting while preserving the BCC-based matrix structure. This is important for subsequent thin-film strain gauge preparation, because a BCC-type and compositionally homogenized multi-principal-element alloy target can help reduce structural and compositional fluctuations during sputtering.

It should be noted that the CALPHAD result represents an equilibrium prediction, whereas the actual phase constitution of the arc-melted and homogenized alloy may also be affected by solidification segregation, diffusion kinetics and cooling rate. Therefore, experimental verification by X-ray diffraction and microstructural characterization is necessary to confirm whether the predicted BCC-dominated phase constitution is retained in the bulk alloy before it is used as a sputtering target.

### 3.2. Phase Constitution and Microstructure of the Bulk HEA Target

**Figure 2:**
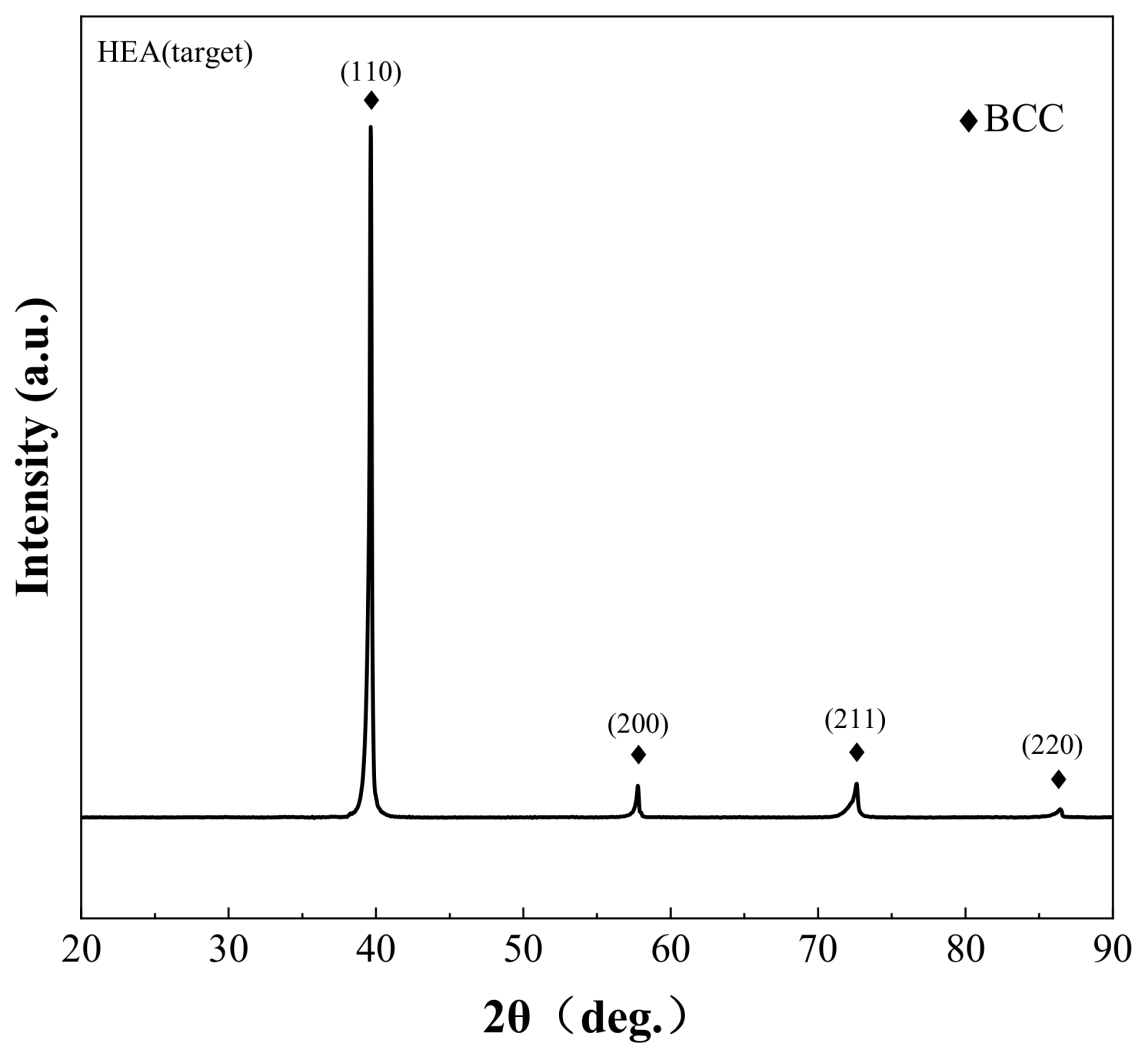
XRD pattern of the homogenized Fe_5_Cu_5_V_30_Ti_30_Nb_30_ bulk HEA target. The main diffraction peaks are indexed as a BCC-type phase, and no obvious secondary phase peaks are detected within the resolution of XRD.

The phase constitution of the homogenized Fe_5_Cu_5_V_30_Ti_30_Nb_30_ bulk alloy was first examined by XRD. As shown in Figure 2, the diffraction pattern exhibits several sharp peaks at approximately 39°, 58°, 73° and 86°. These peaks can be indexed to a BCC-type structure. No obvious diffraction peaks from FCC phases or intermetallic compounds are observed. This result indicates that the alloy target is mainly composed of a BCC-type matrix after vacuum arc melting and homogenization at 1200 °C.

This experimental result is consistent with the CALPHAD prediction of a BCC-based matrix near the homogenization temperature. However, the present XRD pattern does not show resolvable superlattice reflections that would be required to confirm long-range B2 ordering. In addition, conventional XRD may not clearly distinguish a disordered BCC phase from a weakly ordered B2 phase when the superlattice reflections are weak or absent. Therefore, the experimentally observed phase is conservatively described as a BCC-type phase rather than an ordered B2 phase.

**Figure 3:**
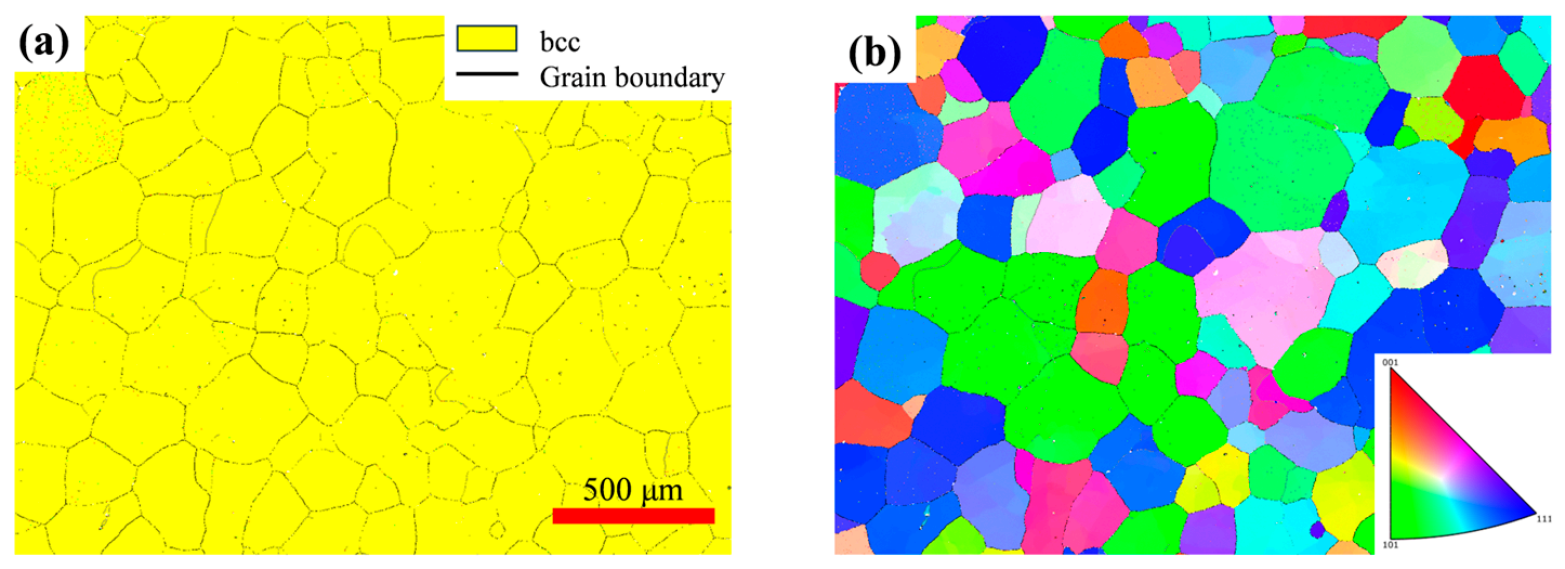
(**a**) EBSD phase map and (**b**) IPF map of the homogenized Fe_5_Cu_5_V_30_Ti_30_Nb_30_ bulk HEA target. Ap-proximately 96% of the scanned area is indexed as BCC, while the remaining ~4% is attributed to zero-solution points (the black spots inside the grains).

EBSD analysis was further performed to examine the phase distribution and grain structure of the bulk target. As shown in Figure 3a, approximately 96% of the scanned area is indexed as the BCC phase. The remaining ~4% corresponds to zero-solution points. These unindexed points are mainly distributed near grain boundaries and at isolated local regions. They are likely related to local surface quality, boundary effects or residual deformation introduced during sample preparation. No continuous secondary phase region is observed in the EBSD phase map. Therefore, the EBSD result confirms that the homogenized alloy is dominated by a BCC phase at the microscale.

The IPF map in Figure 3b shows an equiaxed polycrystalline structure with different crystallographic orientations. A relatively high fraction of green grains is visible in the scanned region, suggesting a possible local preference for <101>-family orientations. Therefore, the EBSD IPF map is used here to describe the grain morphology and local orientation distribution, rather than to claim a random texture. The strong BCC-type (110) peak in the XRD pattern may be related to the intrinsic high intensity of the BCC (110) reflection and/or possible preferred orientation introduced during solidification, homogenization, surface preparation or target machining. Because no pole figure, orientation distribution function or MUD texture index was obtained, the texture strength of the bulk target is not quantified in this work. Together with the XRD result, the EBSD analysis confirms that the Fe_5_Cu_5_V_30_Ti_30_Nb_30_ bulk alloy forms a BCC-type target at the microscale. However, the present data do not provide a quantitative evaluation of texture.

The combined XRD and EBSD results demonstrate that the designed Fe_5_Cu_5_V_30_Ti_30_Nb_30_ alloy forms a stable BCC-type bulk target. This is important for the subsequent thin-film preparation. A phase-uniform and chemically homogenized HEA target can reduce structural and compositional fluctuations during sputtering. It also provides a reliable basis for preparing BCC-type high-entropy alloy thin films for strain gauge applications in high-pressure hydrogen environments.

### 3.3. Structure and Morphology of the HEA Films

**Figure 4:**
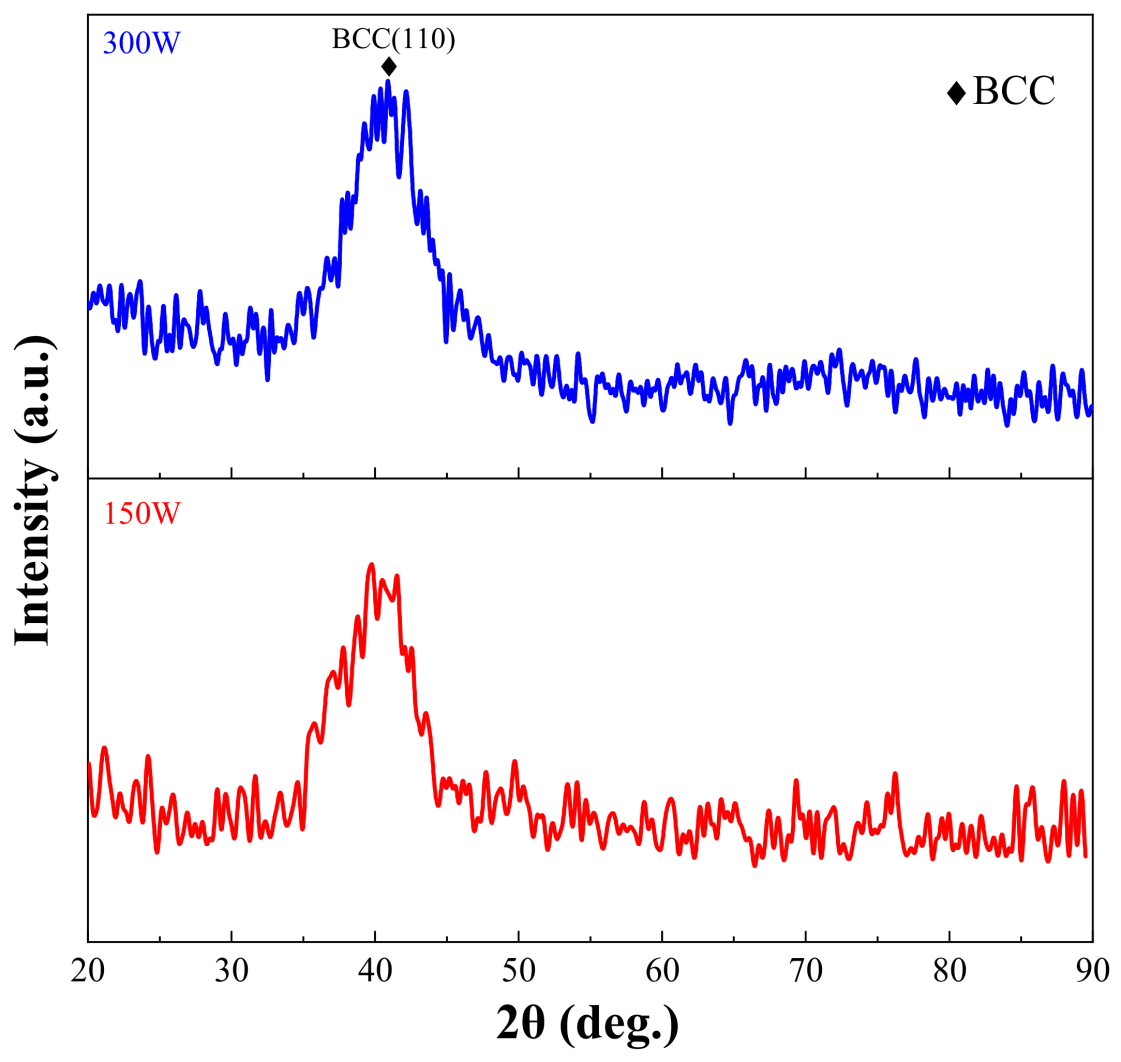
GIXRD patterns of Fe_5_Cu_5_V_30_Ti_30_Nb_30_ films deposited at 150 and 300 W on Si reference substrates. Both films exhibit a broad BCC-type diffraction feature near 40°. The peak broadening is discussed qualitatively only; crystallite size and microstrain are not quantified because only one broad diffraction feature is resolved.

The phase structure of the Fe_5_Cu_5_V_30_Ti_30_Nb_30_ high-entropy alloy films was examined by GIXRD with an incidence angle of 1°. As shown in Figure 4, both films exhibit a broad diffraction peak centered near 40°. This peak is close to the BCC-type (110) reflection observed in the bulk HEA target. No sharp diffraction peaks from secondary crystalline phases are detected. This indicates that the sputtered films mainly retain a BCC-type structure derived from the HEA target. Since no clear superlattice reflections are resolved in the GIXRD patterns of the films, no conclusion regarding B2 ordering is drawn from the film data.

Compared with the bulk alloy target, the diffraction peaks of the films are much broader. This broadening indicates that the films have limited long-range crystallinity. The structure can be described as nanocrystalline or highly disordered BCC-type. This feature is reasonable for sputtered multicomponent alloy films. During sputtering, atoms with different atomic sizes and sputtering yields arrive at the substrate simultaneously. The rapid deposition process limits atomic diffusion and long-range ordering. As a result, the deposited films tend to form a refined and distorted structure.

The peak of the 300 W film is slightly broader than that of the 150 W film, indicating stronger diffraction-peak broadening under the higher sputtering power. Such broadening may originate from several factors, including smaller coherent diffraction domains, microstrain, defect-related broadening, residual stress, lattice distortion and/or highly disordered scattering. However, because only one broad BCC-type diffraction feature is resolved in the present GIXRD patterns and no instrumental-broadening correction, multi-peak line-profile fitting, Williamson–Hall analysis or TEM verification was performed, reliable crystallite-size and microstrain values cannot be extracted. Therefore, the broader peak of the 300 W film is discussed only qualitatively and is not used to draw a definitive conclusion regarding smaller domain size, higher microstrain or greater lattice distortion. It is also not used to establish a direct correlation with the H_2_-related zero-shift behavior of the TFSGs.

**Figure 5:**
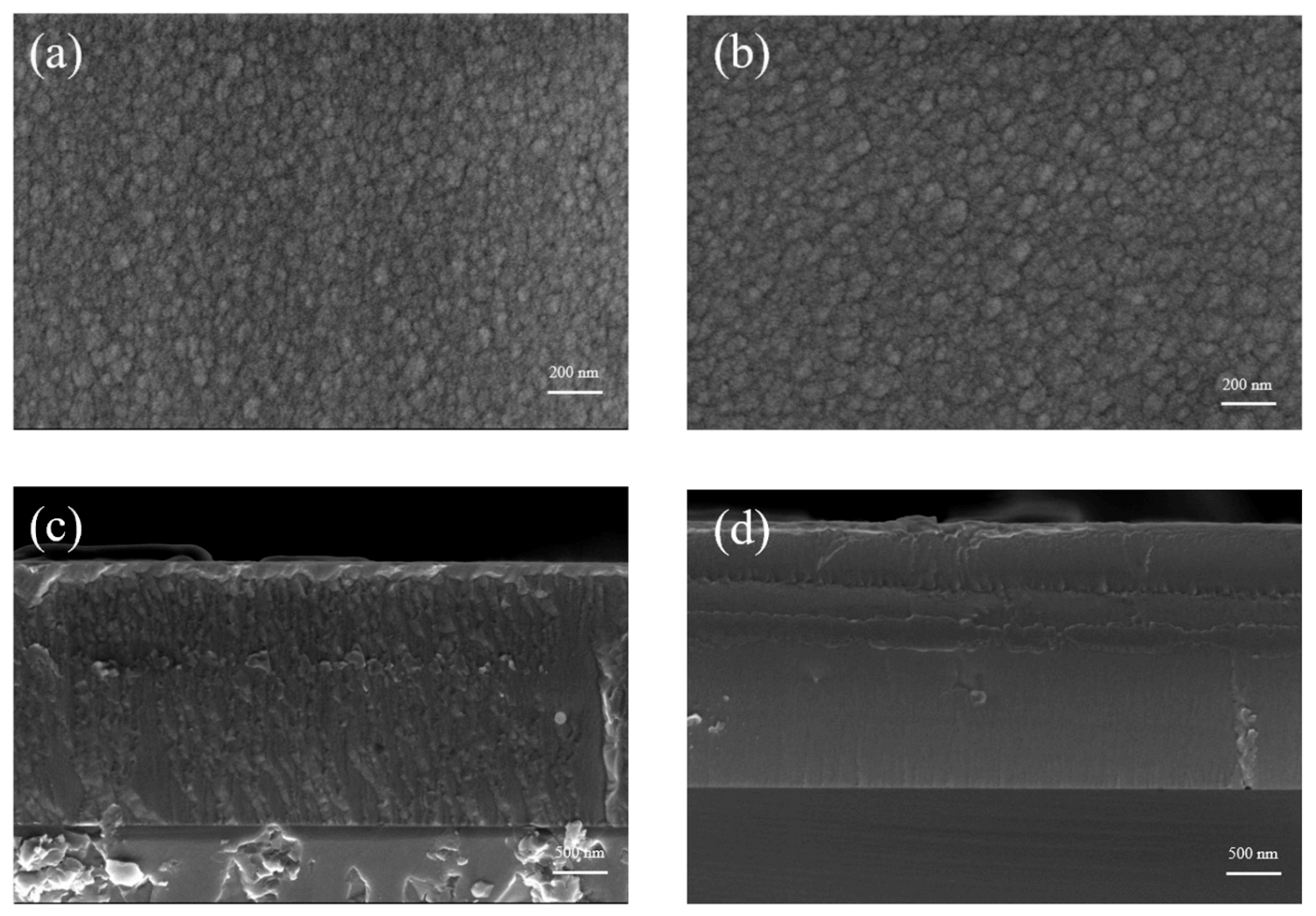
Surface and cross-sectional SEM images of the Fe_5_Cu_5_V_30_Ti_30_Nb_30_ high-entropy alloy films deposited at different sputtering powers: (**a**,**c**) 150 W and (**b**,**d**) 300 W. The surface images show continuous granular morphologies, while the cross-sectional images reveal compact film layers on the substrate.

The surface and cross-sectional morphologies of the films are shown in Figure 5. Both films show continuous surfaces without obvious cracks or large surface defects. The 150 W film presents a fine granular surface, as shown in Figure 5a. The 300 W film also shows a granular morphology, but the surface features are more pronounced, as shown in Figure 5b. This difference indicates that the sputtering power affects the surface growth behavior of the Fe_5_Cu_5_V_30_Ti_30_Nb_30_ films.

The cross-sectional SEM images further reveal the influence of sputtering power on film growth. As shown in Figure 5c, the 150 W film forms a continuous layer with a visible columnar growth tendency. This morphology is usually associated with limited surface diffusion during physical vapor deposition. In contrast, the 300 W film shows a more continuous cross-sectional morphology with less obvious columnar contrast, as shown in Figure 5d. This qualitative difference suggests that increasing the sputtering power may affect the packing and growth mode of the sputtered film, possibly because the higher energy of sputtered species enhances local atomic mobility during deposition. However, the present SEM images do not allow reliable quantitative porosity estimation or columnar-boundary density analysis. Therefore, the morphology comparison is discussed qualitatively only, and no absolute porosity or densification value is reported.

The film thicknesses measured from the cross-sectional SEM images are approximately 2.445 μm for the 150 W film and 2.495 μm for the 300 W film after the same deposition time of 40 min. The corresponding average deposition rates are about 61.13 and 62.38 nm min^−1^, respectively. Although the sputtering power was doubled, the film thickness increased by only about 2.0%, indicating that the growth rate was not linearly proportional to sputtering power under the present deposition conditions. This weak thickness increase may result from the combined effects of higher deposition flux, modified film packing behavior and possible re-sputtering at higher power. Therefore, the similar thicknesses of the two films should not be interpreted simply as a change in deposition rate.

The GIXRD and SEM results together indicate that the sputtered films are continuous and exhibit broad BCC-type diffraction features. The 300 W film shows a more continuous cross-sectional morphology with less obvious columnar contrast than the 150 W film. However, because no quantitative porosity analysis, columnar-density measurement, hydrogen permeation test, or post-exposure structural characterization was performed, no direct causal relationship is established between the observed morphology and hydrogen-ingress resistance. In this work, the film morphology is used only as a qualitative basis for selecting the candidate sensing-layer deposition condition, whereas the hydrogen-environment stability is evaluated at the device level through the subsequent N_2_/H_2_ pressure cycle and strain response tests.

The chemical compositions of the films were estimated by cross-sectional EDS. To evaluate the experimental dispersion, four point/area measurements were performed at different positions on the cross-section of each film. The analyzed positions and the original EDS data are provided in Figure S1 and Table S1, respectively. The averaged compositions and standard deviations are summarized in Table 2. All five designed elements, Fe, Cu, V, Ti and Nb, were detected at all analyzed positions in both films. The 150 W film shows an average composition of Fe_5.05±0.15_Cu_5.66±0.12_V_32.72±0.18_Ti_27.87±0.13_Nb_28.77±0.13_, while the 300 W film shows Fe_5.65±0.15_Cu_4.32±0.12_V_35.83±0.17_Ti_23.49±0.16_Nb_30.65±0.15_. These results indicate that both films retain a refractory-element-rich multi-principal-element composition within the analyzed cross-sectional regions.

Compared with the 150 W film, the 300 W film exhibits relatively higher V and Nb contents and lower Ti and Cu contents, indicating a power-dependent composition-transfer behavior during sputtering. In multicomponent sputtering, the final film composition can be affected by element-dependent sputtering yields, preferential sputtering, angular ejection distributions, gas-phase transport and possible re-sputtering [38,39,40]. However, because no target-surface analysis, angular flux measurement, depth-resolved composition profiling or independent absolute-composition measurement was performed, these factors are discussed only as possible explanations. It should also be noted that SEM-EDS provides semi-quantitative composition estimates. Therefore, the present EDS data are used to confirm the presence of all designed constituent elements and to compare relative composition variations between deposition conditions, rather than to determine definitive absolute film compositions or complete through-thickness homogeneity.

### 3.4. Electrical and Nanomechanical Properties of HEA Films

**Figure 6:**
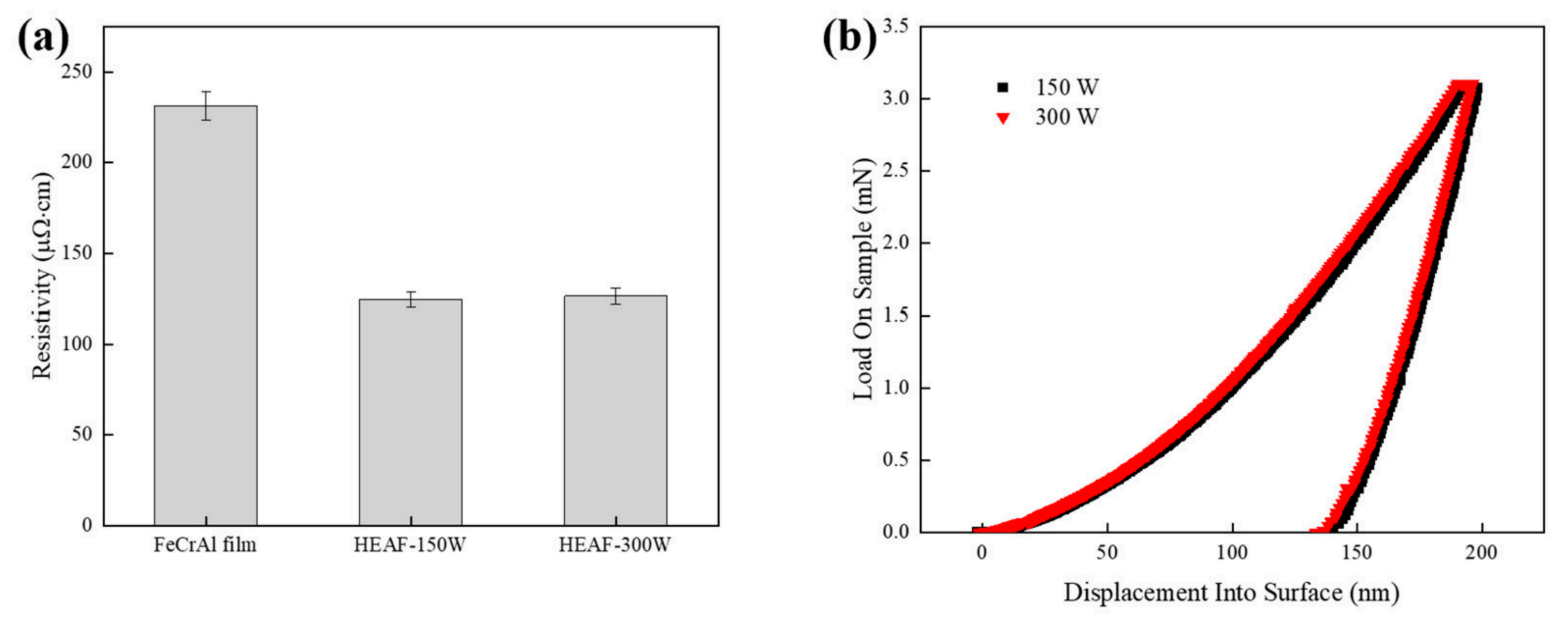
Electrical and nanomechanical properties of Fe_5_Cu_5_V_30_Ti_30_Nb_30_ high-entropy alloy films. (**a**) Electrical resistivity of the FeCrAl reference film and HEA films deposited at 150 and 300 W. Data are presented as mean ± standard deviation (*n* = 5). (**b**) Representative nanoindentation load–displacement curves of HEAF-150W and HEAF-300W selected from five measurements. The maximum indentation depth was fixed at 200 nm.

The electrical and nanomechanical properties of the Fe_5_Cu_5_V_30_Ti_30_Nb_30_ high-entropy alloy films were evaluated to assess their potential as sensing layers for thin-film strain gauges. Figure 6a shows the electrical resistivity of the HEA films deposited at 150 and 300 W, together with that of the FeCrAl reference film. The values are presented as the mean ± standard deviation from five measurements.

The FeCrAl film exhibits a resistivity of approximately 230 μΩ·cm. In comparison, the resistivities of HEAF-150W and HEAF-300W are approximately 125 and 127 μΩ·cm, respectively. The HEA films therefore show a resistivity about 45% lower than that of the FeCrAl reference film. This result indicates that the Fe_5_Cu_5_V_30_Ti_30_Nb_30_ films possess good metallic conductivity and continuous conductive pathways. The resistivity values of HEAF-150W and HEAF-300W are close to each other, indicating that increasing the sputtering power from 150 to 300 W has no obvious negative effect on the macroscopic electrical conduction of the films.

The comparison with FeCrAl is useful because FeCrAl has been used as a sensing layer in previous high-pressure hydrogen thin-film strain gauges [11,14]. However, the lower resistivity of the HEA films should not be interpreted alone as better strain-sensing performance. For resistance-type strain gauges, the device performance is also determined by the gauge factor, temperature coefficient of resistance, long-term drift and environmental stability. The lower resistivity of the HEA films means that a longer grid length or narrower line width may be needed to obtain a comparable initial resistance. Even so, the stable resistivity and small scatter of the HEA films provide a reliable electrical basis for subsequent strain gauge fabrication.

Figure 6b shows representative nanoindentation load–displacement curves of HEAF-150W and HEAF-300W. The maximum indentation depth was fixed at 200 nm. According to the cross-sectional SEM results, the thicknesses of HEAF-150W and HEAF-300W are approximately 2.445 and 2.495 μm, respectively. Thus, the indentation depth corresponds to about 8.2% and 8.0% of the film thickness, respectively. This value is lower than one tenth of the film thickness, which helps reduce the substrate effect during nanoindentation.

Both films show smooth loading and unloading curves. No obvious pop-in event or abrupt displacement burst is observed, suggesting good mechanical integrity during indentation. The hardness and reduced modulus were further extracted from the unloading segments using the Oliver–Pharr method. The hardness values of HEAF-150W and HEAF-300W are 4.80 ± 0.28 and 4.89 ± 0.27 GPa, respectively, while the corresponding reduced moduli are 74.12 ± 0.83 and 72.62 ± 0.65 GPa, respectively. These results indicate that increasing the sputtering power from 150 to 300 W only slightly changes the nanomechanical response of the films. The 300 W film shows a marginally higher hardness, whereas the reduced modulus remains comparable to that of the 150 W film.

This nanomechanical response is consistent with the SEM observations. The 150 W film shows a more evident columnar growth morphology, while the 300 W film exhibits a denser and more uniform cross-sectional structure. The improved compactness of the 300 W film can enhance its load-bearing ability under local contact deformation. This is beneficial for thin-film strain gauges, because a dense sensing layer is expected to improve mechanical continuity and strain transfer during service.

Based on the comparable electrical resistivity, more continuous cross-sectional morphology and slightly improved nanoindentation response, HEAF-300W was selected as the candidate sensing-layer deposition condition for subsequent TFSG fabrication. It should be noted that the above structural, compositional, electrical and nanomechanical characterizations were performed on films deposited on Si reference substrates. These results are useful for comparing the relative effect of sputtering power under controlled deposition conditions, but they should not be interpreted as direct proof that the sensing layer in the final 316L/Cr/AlN/HEA TFSG has identical microstructure or stress state. The 316L/Cr/AlN substrate may influence film growth, strain transfer and residual stress. Therefore, the 300 W condition was selected only as a candidate condition based on the reference-film characterization, and its final applicability was further evaluated by device-level zero-shift and strain response tests in high-pressure gas environments.

### 3.5. Pressure Cycle Zero-Shift Response of HEAF TFSGs in 12 MPa N_2_ and H_2_

**Figure 7:**
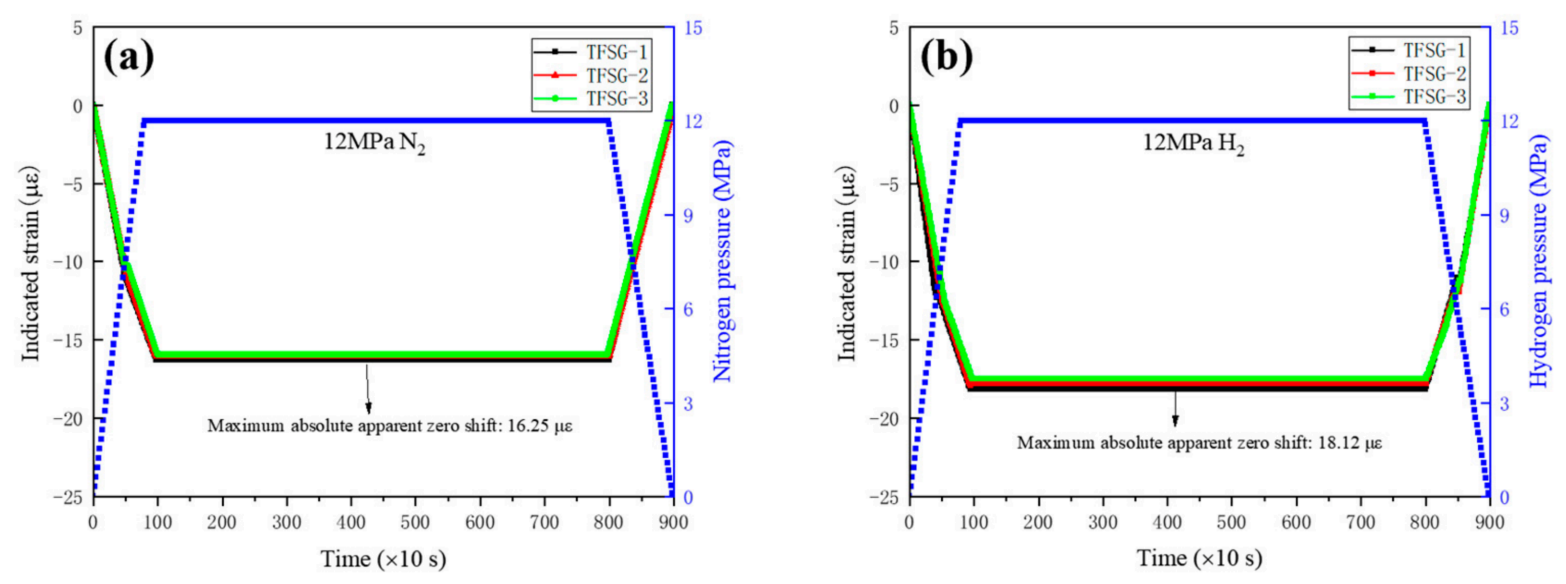
Complete pressure cycle responses of three independently fabricated HEAF TFSGs in (**a**) 12 MPa N_2_ and (**b**) 12 MPa H_2_. The indicated-strain responses are plotted on the left axes, and the corresponding gas-pressure histories are plotted on the right axes. In 12 MPa N_2_, the average and maximum absolute apparent zero shifts were 16.08 and 16.25 με, respectively. In 12 MPa H_2_, the corresponding values were 17.79 and 18.12 με, respectively.

Figure 7 shows the complete pressure cycle responses of three independently fabricated HEAF TFSGs in 12 MPa N_2_ and 12 MPa H_2_. During pressurization, the indicated strain shifted negatively in both gas environments and reached a stable plateau after the pressure was stabilized at 12 MPa. This common negative response in N_2_ and H_2_ indicates that a major part of the apparent output originated from the pressure-induced mechanical/electromechanical response of the substrate–sensor system.

In 12 MPa N_2_, the average absolute apparent zero shift was 16.08 με, and the maximum value was 16.25 με. In 12 MPa H_2_, the corresponding average and maximum values were 17.79 and 18.12 με, respectively. Therefore, the additional H_2_-associated apparent response relative to the same-pressure N_2_ control was 1.71 με based on the average values and 1.87 με based on the conservative maximum values.

During the constant-pressure stage, the outputs of all three TFSGs remained nearly unchanged, indicating negligible time-dependent drift. After depressurization, the indicated strain returned close to the initial zero-pressure baseline. This result suggests that the dominant pressure-induced response was reversible and that the baseline instrumental drift during the pressure cycle was limited.

The previously reported FeCrAl TFSG showed an apparent zero shift of 25.32 με under 12 MPa H_2_ [14]. In comparison, the maximum total apparent zero shift of the present HEAF TFSG was 18.12 με under the same nominal H_2_ pressure, corresponding to a reduction of approximately 28.4%. However, this comparison refers to the total apparent output under H_2_. The hydrogen-related contribution in the present work is more appropriately evaluated from the difference between the H_2_ and N_2_ control tests.

The low total apparent zero shift and the small additional H_2_-associated response may be related to the stable electrical continuity and more continuous morphology of the Fe_5_Cu_5_V_30_Ti_30_Nb_30_ sensing layer. Previous studies have shown that hydrogen dissolution, diffusion and trapping in high-entropy or multi-principal-element alloys can be affected by local chemical complexity, lattice distortion and the distribution of interstitial environments [31,32]. It should also be noted that the hydrogen behavior of BCC Ti-, V- and Nb-containing refractory alloys is highly composition- and microstructure-dependent, and some related alloys have been investigated as hydrogen-sorption or hydride-forming materials [33,34,35]. Therefore, the observed V/Nb enrichment and Ti/Cu depletion in the 300 W film may also influence hydrogen interaction. However, these structural, compositional and morphological factors are discussed here only as possible contributors rather than as experimentally confirmed mechanisms. Because no direct hydrogen absorption, permeation, retention, depth profiling or before-and-after H_2_ exposure characterization was performed, the effect of the composition deviation on hydrogen ingress or hydrogen-related electrical response cannot be separated from the present device-level data. Therefore, the measured response is interpreted as a device-level apparent zero shift under the combined effects of gas pressurization, strain transfer, interfacial stability and possible hydrogen-related electrical perturbation.

### 3.6. Strain-Sensing Performance of HEAF TFSGs in 12 MPa H_2_

To verify that the HEAF device functions as a thin-film strain gauge rather than only as a conductive film with low zero shift, static tensile tests were conducted in 12 MPa H_2_ at 23.0 ± 0.3 °C. The tensile specimen was loaded at a constant displacement rate of 0.05 mm min^−1^, and three independently fabricated HEAF TFSGs were tested under the same condition.

**Figure 8:**
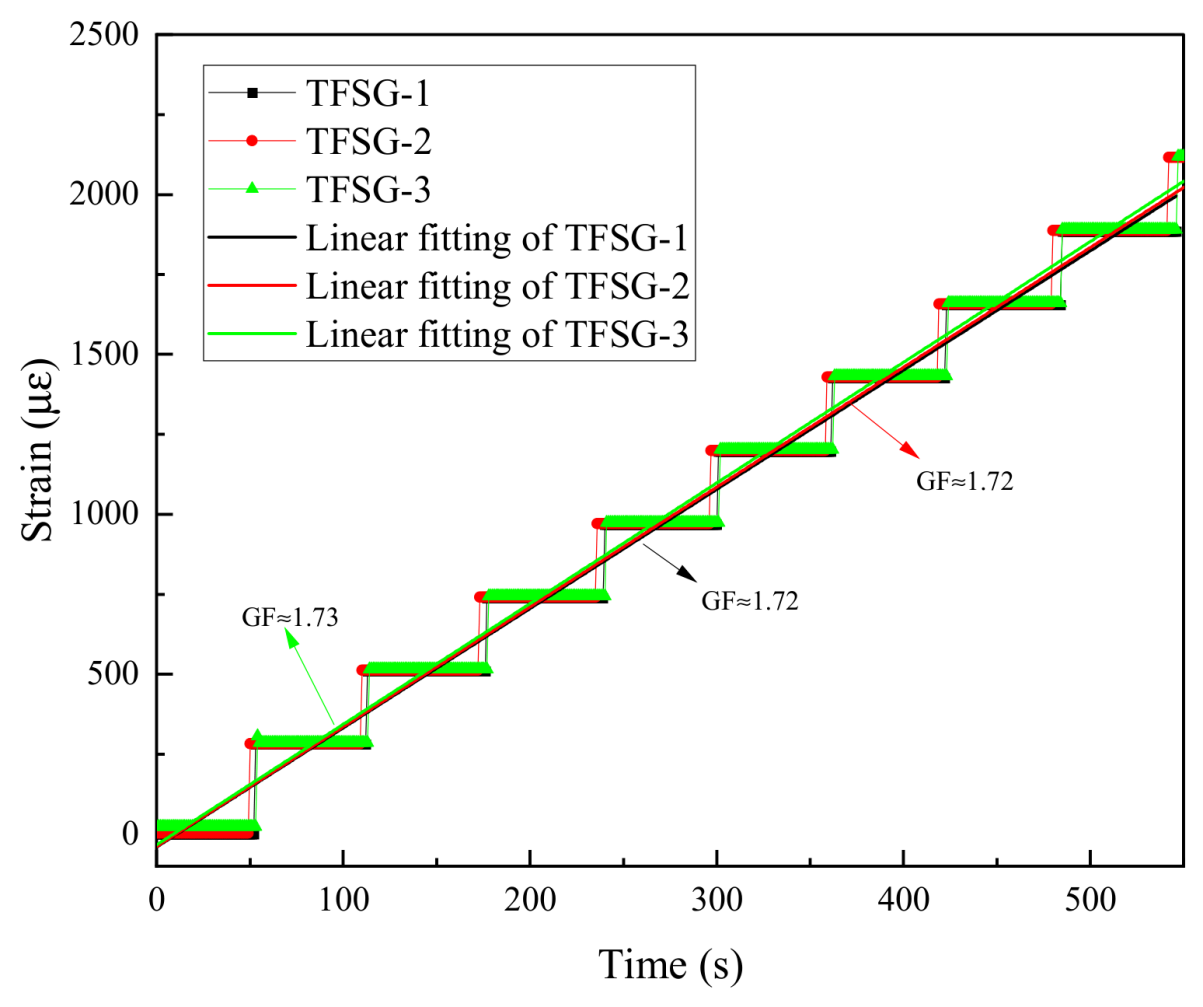
Strain response performance of three independently fabricated HEAF TFSGs in 12 MPa H_2_ at 23.0 ± 0.3 °C under static tensile loading with a constant displacement rate of 0.05 mm min^−1^. Symbols represent the measured responses, and solid lines represent the linear fitting results. After linear-regression normalization, the three datasets nearly overlapped, with R^2^ = 0.9867 and a device-to-device deviation of 0.9%. The calculated gauge factor was 1.72 ± 0.01.

As shown in Figure 8, the output of the HEAF TFSGs increased linearly with loading time under the constant-strain-rate condition. After linear-regression normalization, the three parallel strain–time curves nearly overlapped. The coefficient of determination was R^2^ = 0.9867, and the deviation among the three devices was only 0.9%, indicating excellent repeatability.

The gauge factor calculated using Equation (1) was 1.72 ± 0.01 in 12 MPa H_2_. This value confirms that the Cr/AlN/HEAF multilayer device has a measurable and repeatable resistance response to applied strain in high-pressure hydrogen. Therefore, the present HEAF device is validated not only by its low apparent zero shift, but also by its basic strain gauge performance, including linearity, repeatability, and strain sensitivity.

## 4. Conclusions

In this work, a compositionally defined non-equiatomic Fe_5_Cu_5_V_30_Ti_30_Nb_30_ high-entropy alloy film was investigated as a Cr- and Al-free metallic sensing layer for thin-film strain gauges in high-pressure hydrogen environments. The main conclusions are as follows:

(1) The empirical parameters and CALPHAD calculations indicate that the designed Fe_5_Cu_5_V_30_Ti_30_Nb_30_ alloy has a strong tendency to form a BCC-based matrix. The calculated values, including VEC = 5.15, ΔH_mix_ = −2.69 kJ mol^−1^, δ = 5.29%, Ω = 9.46 and Δχ = 0.086, are consistent with commonly used empirical windows for BCC-type solid-solution formation. The CALPHAD-predicted phase field labelled as BCC_B2 is dominant near the homogenization temperature of 1200 °C, but this prediction is not taken as direct evidence for long-range B2 ordering.
(2) XRD and EBSD results show that the homogenized bulk alloy target is dominated by a BCC-type phase constitution. No clear diffraction peaks from FCC phases or intermetallic compounds are observed, and approximately 96% of the EBSD-scanned area is indexed as BCC. These results indicate that the designed alloy can provide a BCC-type bulk target for subsequent thin-film deposition.
(3) Fe_5_Cu_5_V_30_Ti_30_Nb_30_ films deposited at 150 and 300 W on Si reference substrates exhibit broad BCC-type GIXRD features, suggesting nanocrystalline or highly disordered BCC-type structures. The 300 W film shows a more continuous cross-sectional morphology with less obvious columnar contrast than the 150 W film, but no quantitative porosity or columnar-density analysis was performed.
(4) The HEA reference films show good metallic conductivity, with resistivities of approximately 125–127 μΩ·cm, about 45% lower than that of the FeCrAl reference film. Nanoindentation results show that the 150 and 300 W films have comparable reduced moduli, while the 300 W film exhibits a slightly higher hardness. Because these structural, compositional, electrical and nanomechanical characterizations were performed on Si reference substrates, they are used mainly for comparing sputtering-power effects rather than directly representing the sensing layer in the final 316L/Cr/AlN/HEA TFSG. Accordingly, the 300 W condition was selected as a candidate sensing-layer deposition condition and further evaluated at the device level.
(5) The Cr/AlN/Fe_5_Cu_5_V_30_Ti_30_Nb_30_ TFSGs with the Cr- and Al-free HEA sensing layer exhibited low apparent zero shifts during complete pressure cycle tests. The average absolute apparent zero shifts were 16.08 με in 12 MPa N_2_ and 17.79 με in 12 MPa H_2_, with maximum values of 16.25 and 18.12 με, respectively. The additional H_2_-associated apparent response relative to the N_2_ control was only 1.71 με based on average values. Static tensile tests in 12 MPa H_2_ further confirmed the basic strain-sensing capability of the devices, with a gauge factor of 1.72 ± 0.01, R^2^ = 0.9867 and a device-to-device deviation of 0.9%.

Overall, the Fe_5_Cu_5_V_30_Ti_30_Nb_30_ HEA film shows promise as a Cr- and Al-free metallic sensing-layer material for hydrogen-compatible TFSGs. The present results support its potential for low-drift strain sensing in high-pressure hydrogen, while further work is still needed to quantify substrate-dependent film growth, long-term stability and temperature-dependent sensing behavior.

## Figures and Tables

**Table 1:** Designed composition and calculated thermodynamic parameters of the compositionally defined Fe–Cu–V–Ti–Nb high-entropy alloy.

Alloy Composition	VEC (-)	ΔH_mix_ (kJ mol^−1^)	ΔS_mix_ (J mol^−1^ K^−1^)	Δ (%)	Ω (-)	Δχ (-)	T_m_ (K)
Fe_5_Cu_5_V_30_Ti_30_Nb_30_	5.15	−2.69	11.5	5.29	9.46	0.086	2212.68

Note: Dimensionless quantities are denoted by (-). The alloy is classified as an HEA according to the classical composition-based definition because it contains five principal elements and each element lies within 5–35 at.%. The calculated ΔS_mix_ is approximately 1.38R; therefore, the HEA classification in this work is not based solely on an entropy-threshold criterion. The empirical screening windows used for comparison are −15 ≤ ΔH_mix_ ≤ 5 kJ mol^−1^, δ ≤ 6.5–6.6%, Ω ≥ 1.1 and VEC ≤ 6.87 for BCC-type phase tendency.

**Table 2:** Semi-quantitative cross-sectional EDS composition estimates of Fe_5_Cu_5_V_30_Ti_30_Nb_30_ films deposited at different sputtering powers based on multiple-area measurements.

Sample	*n*	Fe (at. %)	Cu (at. %)	V (at. %)	Ti (at. %)	Nb (at. %)
150 W film	4	5.05 ± 0.15	5.66 ± 0.12	32.72 ± 0.18	27.87 ± 0.13	28.77 ± 0.13
300 W film	4	5.65 ± 0.15	4.32 ± 0.12	35.83 ± 0.17	23.49 ± 0.16	30.65 ± 0.15

Note: Values are presented as mean ± standard deviation based on four cross-sectional EDS point/area measurements. The original EDS data and the analyzed positions are provided in Table S1 and Figure S1.

## Data Availability

The original contributions presented in this study are included in the article/Supplementary Material. Further inquiries can be directed to the corresponding author.

## Supplementary Materials

The following supporting information can be downloaded at: https://www.mdpi.com/article/10.3390/ma19153292/s1, Figure S1. Surface and cross-sectional SEM images of Fe_5_Cu_5_V_30_Ti_30_Nb_30_ high-entropy alloy films deposited at different sputtering powers. Table S1. Semi-quantitative cross-sectional EDS composition estimates of Fe_5_Cu_5_V_30_Ti_30_Nb_30_ films deposited at different sputtering powers based on multiple point/area measurements.
